# Review of grey literature on Ayurveda wound healing formulations and procedures - A systematic review

**DOI:** 10.1016/j.jaim.2023.100779

**Published:** 2023-08-07

**Authors:** Tukaram Sambhaji Dudhamal

**Affiliations:** Department of Shalya Tantra, Institute of Teaching and Research in Ayurveda (Institute of National Importance), Jamnagar, Gujarat 361008, India

**Keywords:** Ayurveda, *Vrana*, *Vranaropana*, Wound, Wound healing

## Abstract

In the era of globalization and evidence-based medicines, a systematic documentation of information by compiling the studies carried out in different parts of India could be useful for the clinicians of Ayurveda and to the ailing community. In this review, an attempt has been made to compile all such clinical research works carried out on *Vrana Ropana* (wound healing). A grey literature of post graduate (PG) and Doctorate (PhD) researches on *Vrana Ropana* from various Ayurvedic institutes were collected in the form of soft or/and hard copy as per the availability. The studies were found to be a combination of drug/drug formulations and various procedures mentioned under *Shashti Upakramas* in Sushruta Samhitha*.* The use of the *Lepa* (topical application), *Avachoornana* (sprinkling of medicated powder)*, Raktamokshana* (bloodletting) like *Jalauokavacharana* (medicinal leech application) and *Kshalana* (therapeutic procedure in which the wounds are cleansed with medicated liquids) were cross reviewed from various research works. All these works were mainly targeted to find the best *Shodhana* (cleaning) and *Ropana* (healing) drugs for the treatment of wound. On the basis of the clinical evidences on the same drug with positive outcomes, one should further try it in multi-centres and develop that drug for wound management. Hence this review study would help to know the previous research works carried out on wound healing and design further trials on specific parameters or treatment protocol as a whole with local as well systemic management of wounded patients.

## 1 Introduction

The ‘art of wound healing’ denotes to surgery and is a major concern to the surgeons. It has been described so extensively in Ayurveda*.* A vast scope of research exists in the field of Ayurveda for the benefit of wound healing. Many herbal and herbo-mineral drugs are being used for wound healing and have proved to be effective to some extent. The formulations used in this system are usually a combination of several compounds that synergistically act together and exhibit multi variant activities [1].

Ayurvedic clinical researches and review articles published in recent years have helped to create a conceptual interface between Ayurveda and modern science in providing significant effects in treating non-healing ulcers and have generated certain leads, emphasizing on the impact of traditional systems of medicine in preventing complications and improving the Quality of life (QoL). Review of such researches and placing them at one place would be beneficial for future researchers and help to generate evidences under single platform.

*Vrana* (Wound/ Ulcer) and healing are the two sides of the surgical coin, on which an expert surgeon has to play his role very sincerely. Since the origin of medicine, there has been a concern about the healing of wounds in general. It is considered to be an essential to life, as it has always been known that infections acquired through wounds might lead to serious complications. Non-healing wounds like diabetic wounds, varicose ulcers, arterial ulcers and pressure ulcers are chronic wounds that are reluctant to heal [2]. All these wounds hamper the quality of life (QoL) of patients. In conventional surgery, the treatment of these chronic wounds are antibiotics and dressing with antiseptics which have their own limitations and adverse effects.

Current scenario needs to have evidences on *Ayurvedic* treatments. Development of a system always depends upon the quality publications of the research works. However, attempts on compiling all such research publications on a specific disease (wounds) are very rare [3]. Accordingly, herein efforts have been made to compile all the unpublished research works or grey literature on Ayurveda wound healing formulations and procedures in the structure of systematic review.

### 1.1 Objective of study

To summarize the clinical evidences on wound healing through grey literature of Ayurveda research works conducted in Ayurveda teaching institutes of India.

### 1.2 Study centre

The project “Systematic reviews on *Vrana Ropana* (wound healing) properties of Ayurvedic Drugs” was conducted at Institute for Post Graduate Teaching and Research in Ayurveda (IPGT&RA- A WHO collaborating Centre for traditional medicine), Jamnagar, Gujarat Ayurveda University (Accredited grade ‘A’ NAAC) through internal grant of the institute as intra mural project (IMR). Now it is known as Institute of Teaching and Research in Ayurveda (Institute of National Importance) w. e.f. 2020.

### 1.3 Searching

**Fig. 1:**
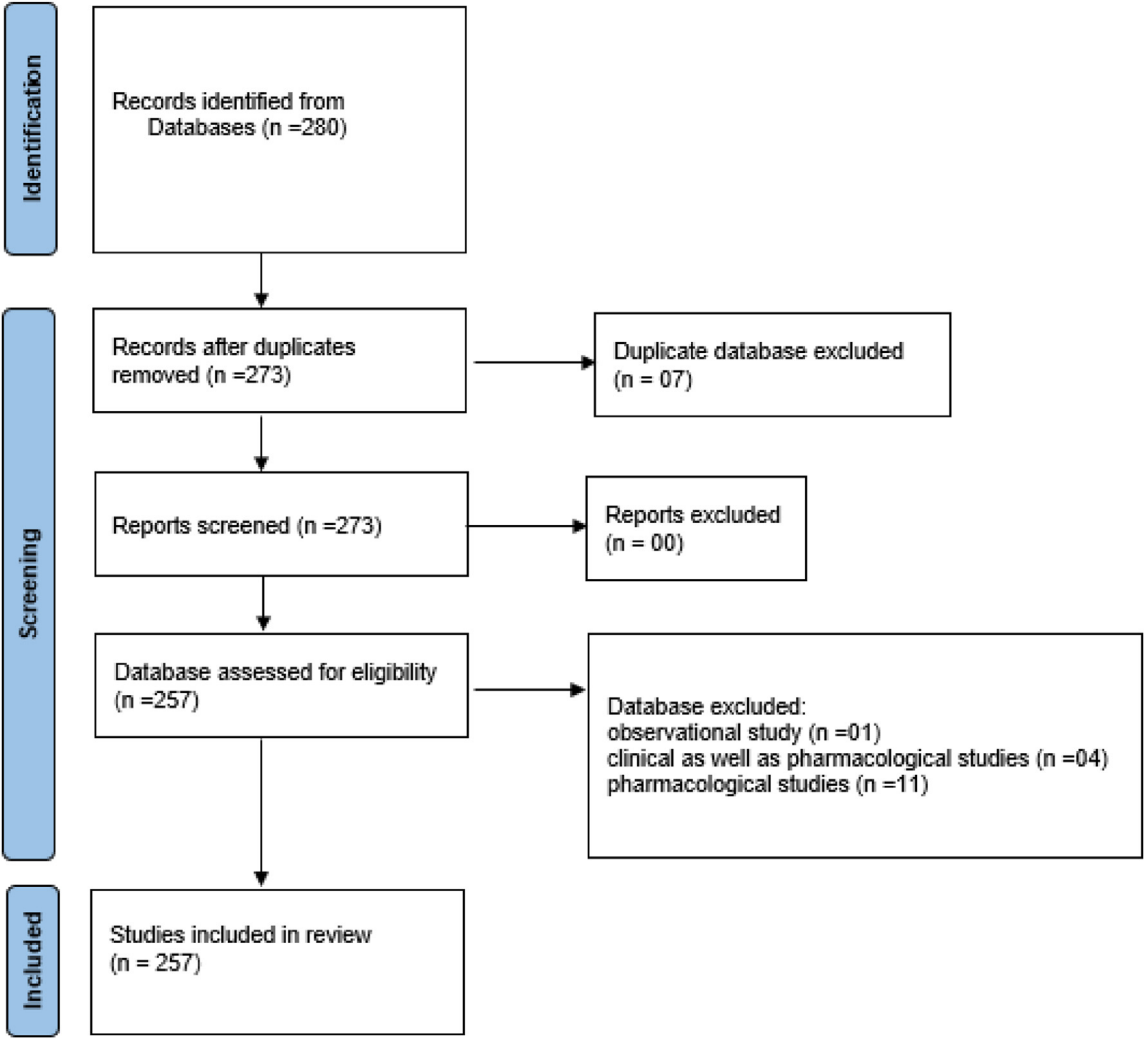
Flowchart illustrating the selection of the 257 trails included in systemic review.

Total 273 thesis were searched by personal visits (hand search) to Ayurveda PG institutes attached to health universities or Ayurveda universities of India. Among them only 257 clinical studies were included in the review as per inclusion criteria of research protocol [Table 1, Table 2] (Fig. 1). The search was done by junior research fellow (JRF)/ Senior research fellow (SRF)/ principal investigator (PI) of the IMR research project as per assigned from time to time. The clinical works done during 1971–2016 were found/included in this review on wound healing.

**Table 1:** Indian health or Ayurveda Universities approached for data collection.

Name of the University	State
Gujarat Ayurved University, Jamnagar	Gujarat
Dr. Sarvapalli Radhakrishnan Ayurved University, Jodhpur	Rajasthan
Banaras Hindu University, Varanasi	Uttar Pradesh
Rajiv Gandhi University of Health Sciences (RGUHS), Bangalore	Karnataka
NTR University of Health Sciences, Vijayawada,	Andhra Pradesh
Maharashtra University of Health Sciences (MUHS), Nashik,	Maharashtra
Kerala University of Health Sciences, Thrissur	Kerala
Ayush & Health Science University, Raipur	Chhattisgarh
Himachal Pradesh University, Shimala	Himachal Pradesh
Bharati Vidyapeeth Deemed University, Pune,	Maharashtra
Uttarakhand Ayurved University, Dehradun	Uttarakhand
KLE Deemed University, Belgaum	Karnataka
Dr. Babasaheb Ambedkar Marathwada University, Aurangabad	Maharashtra
Swami Ramanand Teertha Marathwada University, Nanded	Maharashtra

**Table 2:** Types of studies.

Type of studies	No. of studies reported
Clinical as well as Pharmacological studies	4
Pharmacological studies	11
Observational study	1
Clinical studies only	257

## 2 Study selection

### 2.1 Inclusion criteria

1. Grey literature from Ayurveda research institutes having clinical trials on *Vrana Ropana* (wound healing) were included in this review.
2. Clinical trials on local wound healing drugs/interventions/procedures/ para-surgical/ *Panchakarma* procedures were included in this review.
3. Clinical trials on local as well as oral systemic drugs tried for *Vrana Ropana* (wound healing) were also included in this review.

### 2.2 Exclusion criteria

1. Clinical trials on herbal drugs conducted on places other than Ayurveda institutes were excluded.
2. Pharmacological studies carried out on Ayurveda drugs were excluded
3. In-vitro studies on Ayurveda drugs were excluded from this review.
4. Published online papers on wound healing Ayurveda drugs were also excluded from this review.

### 2.3 Outcomes assessed in the review

1. Which Ayurveda drug or formulation is effective in management of wounds?
2. Which para-surgical procedure is effective in wound care along with oral or local management?
3. Which *Panchakarma* procedure is suitable for wound healing?
4. What is the end outcome of the clinical studies carried out in different Ayurveda teaching institutes?

### 2.4 Data extraction

In the first three months, the format of abstract to fill all the clinical trials were prepared. The request letter was sent to all principals/deans/heads of the institutes for permission to search the research work in their respective institutes. The person visited the principals of concerned institutes and after permission the data on wound healing were extracted from the thesis available in the institutional library (Grey literature). All the related informations especially the clinical studies were scanned and collected in pen drive or external hard disc for further analysis. In reference to Prof. MS Baghel's book on Researches in Ayurveda, the search was conducted manually [4].

### 2.5 Method of analysis of collected research data

The collected or extracted data on wound healing from different institutes have been summarized in the specially designed format. This format includes the title of the research work, methodology adopted, interventions details, assessment parameters, observed results and concluding remarks of the concerned researcher [5].

## 3 Result of the review

### 3.1 Local application of Ayurveda herbal drugs

The data collected in this review reveals numerous Ayurvedic formulations used for wound healing. In this review, research works reveal total 220 different formulations that have been used for wound healing. For wound healing, *Jatyadi Taila* [6] and *Jatyadi Ghrita* was used for healing in 19 and 13 research studies respectively. 15 studies have been carried out on different forms of *Nimba* (*Azadirachta indica* A. Juss). *Panchavalkala* (five plant barks combination) was used in various dosages forms like *Kwatha* (Decoction), *Churna* (powder), *Malahar* (cream), *Kshara* (Plant Alkali), *Raskriya* (concentrated extract), *Taila* (medicated oil), *Ghrita* (medicated ghee). *Yashtimadhu* (*Glycyrrhiza glabra* Linn.) which is one of the good healing herbs was also tried in forms like *Churna* (Powder), *Kalka* (paste), *Taila* (medicated oil), *Ghrita* (medicated ghee). Total 9 researches have been conducted on plain *Madhu* (honey) while 11 researches were conducted on *Madhu* along with additional single drug. In this review, it was also observed that povidone iodine ointment and povidone iodine solution were used as control group in 37 and 12 research studies respectively.

### 3.2 Procedures for wound management

In review, 24 different types of para-surgical and *Panchakarma* procedures were conducted for wound healing. Among them, 17 research works were on *Jaloukavacharana* (Medicinal leech application), 4 studies were conducted on *Agnikarma* (Therapeutic heat application) in cervical erosion. *Virechana Karma* (Therapeutic purgation), *Matrabasti* (Medicated enema), *Vrana basti* (Putting medicated oil in wound), *Dhoopan karma* (Fumigation with medicinal herbs) and *Siravedha* (Therapeutic vein puncture) were also mentioned in the studies.

### 3.3 Types of wounds

In acute wounds, 42 research studies were done on *Sadyovrana* (acute postoperative or traumatic wounds) and 2 studies on episiotomy wounds. In context to wound types, studies on wounds at anal region i.e., 13 studies were on fissure-in-ano, 12 studies were on *Dagdha vrana* (burn wounds) and 12 were on traumatic wounds. Out of 156 studies conducted on chronic ulcers, 119 studies were on *Dushta Vrana* (Non-healing wounds or chronic wounds) while on specific wounds, 19 studies were on diabetic wounds.

### 3.4 Types of studies

52 studies were conducted as single arm type studies, 96 studies were comparative studies and 86 researches conducted as randomized controlled trial (RCT) in this review.

### 3.5 Types of interventions

Most of the studies involved multiple formulations as comparative groups while few studies attempted on evaluating efficacy of single formulation or single drug in the management of wounds. Some studies attempted on evaluating the impact of *Panchakarma* procedures on *Dushta Vrana* [7]. Some studies were carried out by using para-surgical procedures like *Agnikarma* and *Ksharkarma* along with internal oral medications [8] [Table 3] The studies were found to be a combination of drugs/drug formulations and the procedures mentioned under *Shashti Upakrama* (sixty measures for wound healing) by *Acharya Sushruta* [9]. The aim of the management of wounds was the removal of contaminated, devitalized tissues or necrosed tissues and foreign bodies, to achieve adequate haemostasis and apposition of the wound, to prevent infection and complications to initiate early healing. It was observed that the use of the *Lepa*, *Avachoornana, and* para-surgical procedures *Raktamokshana* such as *Jalauokavacharana* [10,11] and few other procedures like *Kshalana* reported positive outcome in wound healing [12]. Some of the studies were intended on *Agnikarma* and *Ksharakarma* in *Dushta Vrana* with encouraging results [13].

**Table 3:** Different para-surgical procedures adopted.

*Agnikarma*-1	*Jalaoukavacharana*- 9
*Raktamokshana*-1	*Ksharakarma* + *Agnikarma*-1
*Avachoornana* + *Agnikarma*-1	*Ksharasutra*-1

### 3.6 Compound/poly-herbal preparations used for wound healing

17 single drug formulations and 10 combination drug formulations were studied in this review [[14], [15], [16], [17], [18], [19], [20], [21], [22]]. Compounds or poly herbal dosage forms were used in many of the research studies or thesis. In five studies, *Kalka* was used for dressing, among them *Tila Kalka* [23] was used the most along with other formulations like *Nimba Patra Kalka* and *Yava Kalka*. *Kwatha* like *Dasamula Kwatha* [24]*, Bhadramusthati Kwatha* [25]*, Dhanwanthara Kwatha, Patoladi Kwatha* [26] were used to clean the wound in addition to the conventional use of *Pachavalkala Kwatha* [27] and *Triphala Kwatha* [28]. The formulations of *Avachoornana* such as *Trivritadi Churna* [29], *Guggulu Panchapala Churna* [30] were studied in various research works. The base for majority of topical applications were cow *Ghee, oil* and *Malahara* [31]. *Jatyadi Ghrita, Yashtimadhu Ghrita* [32], *Manjishtadi Ghrita* [33] and *Durva Ghrita* [34] were used in most of the *Ghrita* preparations [35,36] and among *Taila* preparations *Kampillakadi Taila* [37] was studied the most [38]. *Dhoopana* drugs such as *Nimbadi Dhoopana* [39] and *Guggulu Dhoopana* was evaluated in research works for their wound healing activity [40,41]. *Panchavalkala Rasakriya, Dwiharidra Rasakriya* [42]*, Yasada Bhasma, Swarna Makshika Bhasma* etc. were used too by researchers for wound healing [Table 4].

**Table 4:** Compound Formulations used in research studies.

*Kalka* [Medicated paste]– 5	*Kwatha* [Medicated decoctions] – 8
*Swarasa* [Medicated Juice]-1	*Choorna* [Powder form]- 18
*Ghrita* [Medicated ghee] - 34	*Taila* [Medicated oil]- 43
*Dhoopana yoga* [Medicated fumigation] - 4	*Rasakriya* [Medicated concentrated decoction]-4
Folklore Medicine [Non-classical medications]- 2	*Guggulu* [Medicated resins]-6
*Kshara* [Plant alkalies]- 8	*Arka* (Medicated extract)-1
*Malahara* [Medicated ointments] - 9	*Lepa* [Medicated liniments]- 16
*Varti* [Medicated wick]- 6	*Vati* [tablets]- 3
Bhasma- 4	*Panchakarma* or para-surgical procedures
*Ksheera* [Latex]-4	Single Drugs-17
Compound drugs-10	

### 3.7 Pharmacological action of the drugs

Wounds are of two types, intrinsic and extrinsic. The intrinsic one is caused by the vitiation of *Vata, Pitta, Rakta* and *Sannipata* while the extrinsic wound is caused by human beings, animals, birds, ferocious beasts, reptiles, falls, pressing, fire strike, caustic, alkali, poison, irritant drugs, pieces (of wood etc.), earthen ware, circular weapons, arrow, axe, trident, spear etc. In exogenous wound in initial stage there is no involvement of *Doshas* but later on leads to vitiation of *Doshas*. Majority of topical dosage forms used in these research works had ghee, oil and *Malahara* base which symbolizes that they facilitate enhanced drug penetration for early wound healing [43]. In fresh wound formulations applied externally like *Durva Ghrita, Durva Taila,* [44] *Madhuyashtyadi Ghrita, Talpatri Ghrita, Karanjadi Ghrta* [45]*,* and *Taila* formulations like *Manjishtadi* [46]*, Chandanadi* [47]*, Vranaropan, Kampillakadi, Noola Taila* etc and *Madhu Ghruta* [48,49] equally showed effective as healing agents.

On analysing the research studies, it seems that all studies mainly were targeted to find a best *Shodhana* (cleansing) and *Ropana* (healing) drug for wound management [50]. The drugs used in the research works have therapeutic properties like *Lekhana (*scraping)*, Shoshana* (emaciating)*, Shodhana (*purification*), Kledahara* (emulsification)*, Krimighna (*anti-helminthic*)* with *Kashaya (*astringent*), Tikta (*bitter*), Madhura Rasa (*sweet*), Ushna Virya* (hot potency) and *Teekshna Guna (*penetrating)*.* Majority of drug preparations has tannins with phytosterols which reduces secretions and acts as good emulsifiers. Tannins & Phytosterols promote the healing process by wound contraction with increased capillary formation and fibroblasts proliferation followed by enhancing the rate of epithelialisation [51]. Preparations having ghee, oil and *Malahara* base facilitate enhanced drug penetration for early wound healing providing nutrition to the tissue as moist dressing [52].

Most of the studied drugs possess the properties like, *Lekhana* (scrapping), *Shoolahara* (analgesic), *Amapachana* (digestive), *Kapha Vata Shamana*, *Kledashoshaka* (drying up), *Sthambhana* (haemostasis or reduce discharges)*, Jantughna* (antibacterial), *Varnya* (bring normal skin color) property checks the *Vrana Varna* (color of wound and surrounding tissue), *Vedana* (pain at wound site), *Gandha* (oudor), *Srava* (wound discharge), and removes the slough in the *Vrana* [53,54]. Those drugs also have *Ushna Veerya* (hot potency), and probably it stimulates the proliferation of healthy granulation tissue [55]. The *Lekhana* (scraping) *Guna* of the formulations tried in research work helped to debride the slough and unhealthy granulation tissue which promoted healing [56]. The *Shoolahara* (analgesic) *Guna* pacified the pain, due to *Amapachana* (digestion) the local unprocessed metabolites were cleared and there by increased the local blood circulation [57]. Due to *Kapha-Vata Shamaka*, *Ruksha* (dryness) and *Kledashoshana* (drying up) *Guna*, the secretion and local infection were cleared [58,59]. The *Jantughna* (antibacterial) properties of the drugs took care of the local infection. On the basis of different research analysis done over years, these drugs/drug formulations have antiseptic, anti-inflammatory, prostaglandin inhibitor, anti-histaminic and anti-microbial properties [[60], [61], [62]].

## 4 Discussion on various/different dosages forms

In Ayurveda, seven dosages forms have been mentioned by *Acharya* Sushruta like *Kashaya* (Decoction), *Varti* (Wick), *Kalka* (Paste), *Sarpi* (Clarified butter) *Taila* (medicated oil), *Rasakriya* (Semi solid extract), *Choorna* (Powder for dusting). The rationale behind this is when these forms are being used one should know the *Doshic* involvement in the wound, duration of the wound, depth of the wound, amount of exudate, stage of the wound, patients *Prakriti*. If these guidelines are followed then the patients can be recovered earlier from symptoms and promote early wound healing.

### 4.1 Discussion on different treatment

The rationale behind individualised treatment for wound management in Ayurveda instead of a common treatment for all patients is the assessment of the wound status (tissue viability, presence or absence of slough, exudates etc) and *Prakrithi* of the patient. In wound management also, if patient is suffering from diabetic foot ulcer and if there is atherosclerosis then along with local wound management leech therapy will be more helpful as well as systemic management of diabetes mellitus also becomes a part of the treatment.

Discussion should include statements on findings of different treatment.

### 4.2 Merits of systematic review

1. The grey literature is not exposed to further research due to lack of open access. So with this review it is possible to get access of the drugs tried in clinical studies for wound healing.
2. Researcher or scholar can design new research studies in a greater number of cases on the basis of these pilot studies carried out in small sample size.
3. It will create or generate the interest to work on wound healing through Ayurvedic interventions in young scientist/ researchers involved in wound healing speciality.
4. Many studies are being conducted on wound healing but due to unpublished data it does not come into practice. Consequently, this review gives knowledge and exact evidences about wound healing through Ayurvedic drugs and procedures.

### 4.3 Limitations of systematic review

1. Outcome measures of the studies are different though the same interventions have been tried in the study.
2. The protocol designed for the research studies vary from institute to institute.
3. The assessment of the result was done with the help of Ayurvedic and modern parameters and duration of treatment varied from study to study.
4. Assessment criteria different from study to study though similar disease condition was being treated by two researchers.

On the basis of this review the non -healing wounds like diabetic wounds, venous leg ulcers, arterial ulcers and pressure ulcers can be well managed with Ayurvedic medications along with integrated approach. Diabetic foot management can be done with local application of wound cleansing and wound healing agents. In non-viable tissues, local debridement with K*shara*, off-loading with POP, *Jaloukavacharna* (leech application) in atherosclerosis and foot care as well as glycaemic control by systemic Ayurvedic medication can heal the diabetic wounds and save from amputations. In venous leg ulcers, particularly in eczema, blood-letting (*Siravedha*)*, Jaloukavacharna* (leech application)*,* compression stockings, limb elevation, calf muscle exercises along with life style modifications can stop the further progress of varicosities and wound healing takes place. In arterial ulcers, the cause of arterial blockage needs to be addressed during wound management. In TAO, the management includes local *Abhyanga* and *Nadiswedan, Jaloukavacharan*, and strong analgesic or vasodilators whenever needed. The pressure ulcers are usually non-healable unless and until the cause of the pressure is not addressed. In such type of ulcers, local wound dressing, nutrition as well as nursing care plays an important role. In a nutshell, it can be said that with holistic approach, non-healing wounds can be managed with Ayurveda medicaments.

## 5 Conclusion

A suitable drugs list or drug formulations for the management of *Vrana* would stand against the commonly used medicaments. This review has considered many classes of drugs or drug formulations dealt with many research works undergone in different research institutions of India. It is significant to note that each Ayurvedic formulation or single compound has its own mechanism of action that individually stimulates wound healing.

In the era of globalization and evidence-based medicines, this systematic documentation of such information by compiling the studies from different parts of the country will benefit the researcher for further critical analysis and to design new studies. This review suggests that wound healing using Ayurvedic drugs and procedures are safely in different dosage forms. This systematic review also creates a concrete platform for future researchers and Ayurvedic practitioners where unpublished trials are easily availed with regards to Ayurvedic wound healing treatment.

### 5.1 Strategies

Efforts should be undertaken to identify potent medicines derived from wound healing clinical trials conducted at nationally recognized Ayurveda institutes. Furthermore, it is crucial to encourage other institutes throughout the country to conduct multicentric research on these drugs while maintaining uniformity in their methodologies. Based on the evidence generated, policymakers can motivate pharmacological companies to produce these drugs or formulations on a larger scale and distribute them nationwide. So that clinically tested or proved wound healing drugs or formulations can practice and outreach to non-academician practicing doctors.

Meta-analysis poses considerable challenges within the field of Ayurveda due to the scarcity of proper and uniform clinical trials throughout India. The majority of trials feature inadequate sample sizes, typically ranging from 20 to 40 patients. The research methodology lacks uniformity as well. Such sample sizes are insufficient for conducting meta-analyses and generating substantial evidence. To facilitate the substitution of Povidone Iodine with Ayurvedic formulations at the grassroots level, a uniform, structured, dedicated, and multidisciplinary drug development system is imperative.

It is crucial to refrain from approving any clinical studies in Ayurveda without the availability of preclinical data. The clinical trials should adhere to phases 1–4. However, completing all these phases within the span of a three-year postgraduate program is unfeasible. So collabortive work with CCRAS and other research councils and phram company is essential to develop the drug and make it available in the market for easily avialble through out India.

## Funding

Institute for intra-mural research project of Institute for Post Graduate Teaching and Research in Ayurveda (IPGT&RA) as IMR project Ref. No. PGT/TRC/2010–2011/66, dated 08-04-2011.

## Author contribution

Dudhamal TS: Protocol design, data collection, manuscript writing, reference collection and final approval.

## Declaration of competing interest

None.
